# Plantar Fasciitis With a Calcaneal Spur

**DOI:** 10.7759/cureus.51242

**Published:** 2023-12-28

**Authors:** Shiva Sajja, Nubaha Elahi, Latha Ganti

**Affiliations:** 1 Biology, The Walker School, Marietta, USA; 2 Emergency Medicine, Maimonides Medical Center, Brooklyn, USA; 3 Medical Sciences, The Warren Alpert Medical School of Brown University, Providence, USA; 4 Emergency Medicine and Neurology, University of Central Florida College of Medicine, Orlando, USA

**Keywords:** talalgia, foot pain, heel spur, calcaneal spur, plantar fascitis

## Abstract

This study presents the case of a 47-year-old male with right foot plantar fasciitis and a calcaneal spur. Chronic heel pain can be caused by several medical conditions, including plantar fasciitis and a calcaneal spur, which often may be overlooked on initial evaluation. The risk factors, clinical presentation, imaging findings, and emergency department management of plantar fasciitis with a calcaneal spur are reported and discussed.

## Introduction

Calcaneal spurs are bony projections that form around the heel bone (calcaneus), the strongest and posterior-most bone in the foot [1]. The classic symptom of a calcaneal spur is heel pain, also known as talalgia [1]. They are frequently associated with plantar fasciitis [2]. Plantar fasciitis is a painful inflammation of the connective tissue (plantar fascia) that extends along the bottom of the foot and connects the heel bone to the ball of the foot. Risk factors for heel spurs include gait abnormalities which place excessive stress on the heel bone and ligaments, excessive running or jogging, poorly fitted shoes, or excess weight gain and obesity.

Heel pain with a calcaneal spur can sometimes be mistaken for other medical conditions such as Baxter’s nerve compression, fat pad atrophy, Achilles tendinitis, stress fractures of the calcaneus, tarsal tunnel syndrome, and lumbosacral spine radiculopathy, all of which may be part of the differential diagnosis.

## Case presentation

A 47-year-old male presented to the emergency department with chronic right heel pain. The pain was worse in the morning but improved as the day progressed. Symptoms had been ongoing for several weeks. The patient reported no trauma to the fifth metatarsal. Two weeks prior, he was seen by a physician; the right foot was examined, and the X-ray revealed no acute abnormality or fracture. The patient had been seen by a chiropractor for his right heel pain. The chiropractor scraped and manipulated his foot, but in the long term, there was no improvement; in fact, symptoms worsened. The patient denied any tobacco use and had no known drug allergies.

**Figure 1:**
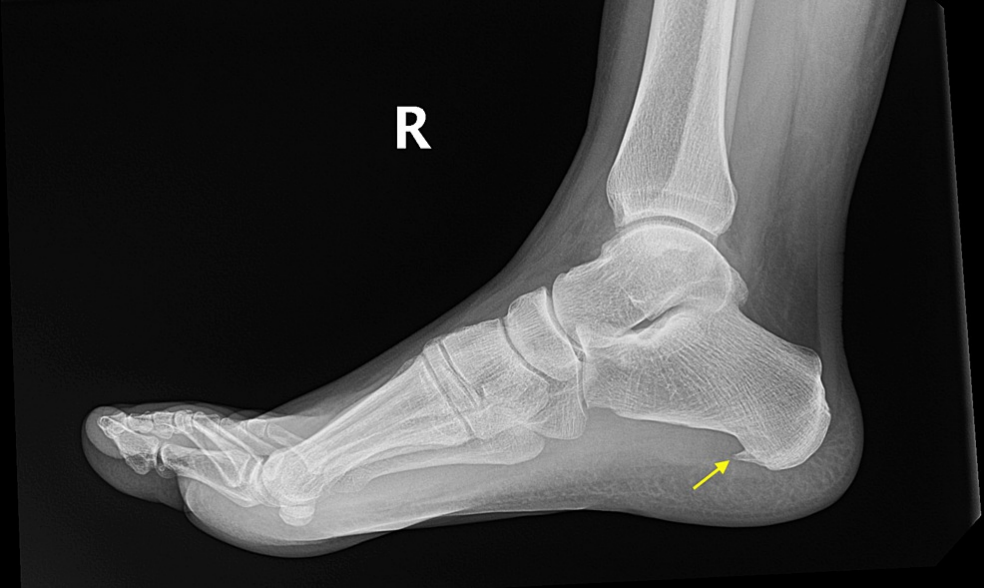
Radiograph depicting a calcaneal spur (arrow)

On physical examination, the vital signs revealed a temperature of 97.9 degrees Fahrenheit, a blood pressure of 129/90 mmHg, and a heart rate of 61 beats per minute. Respiration was 16 breaths per minute, and oxygen saturation was 100% on room air. A focused physical exam revealed a well-appearing male in no acute distress. The lower extremity, pelvis, and musculoskeletal system were atraumatic, with no acute findings. No abnormalities were seen on the neurological and vascular exams. There was no ligament or tendon injury seen, and the gait was normal. No findings of compartment syndrome, signs of edema, or circumferential injury were noted. The right ankle and foot physical exam demonstrated a full range of motion without swelling or erythema. There was no deformity appreciated, and the foot was non-tender to touch. The radiograph revealed a calcaneal spur (Figure 1).

The patient was diagnosed with a calcaneal spur and right plantar fasciitis. He was sent home with specific instructions for exercise and strengthening of the right foot. This included exercises such as picking up marbles and pencils with his toes. The patient was also advised to stretch his calf and Achilles tendon by rolling the affected foot over a bottle. Arch support, massage, and non-steroidal anti-inflammatory medications were recommended to assist with analgesia. The patient was instructed to avoid walking barefoot and to use supportive shoes. He was discharged with a final diagnosis of right plantar fasciitis and a calcaneal spur, with instructions to follow up with his primary care physician for further medical care.

## Discussion

Calcaneal spurs can be located on the dorsal aspect of the calcaneus as well as the plantar surface. They are calcium deposits causing a triangular bony protrusion on the bottom of the heel bone. On plain radiographs, they can sometimes protrude by as much as half an inch. While most are painless, some can cause heel pain. Heel spurs usually occur as a result of strains on foot muscles and ligaments, stretching of the plantar fascia, and repeated tearing of the membrane that covers the heel bone. Athletes are at risk due to excessive running and jumping. Additionally, age can increase the risk of developing plantar fasciitis with calcaneal spurs because the plantar fascia becomes less flexible over time. Age can also lead to a decrease in the natural fat pad surrounding the calcaneal bone. Individuals with flat feet or high arches are at an increased risk of developing plantar fasciitis with a calcaneal spur. Calcaneal spurs are associated with various medical conditions, including obesity, plantar fasciitis, rheumatoid arthritis, osteoarthritis, ankylosing spondylitis, Reiter's disease, and spondylarthritis [3-5].

Several factors should be considered when analyzing the impact of a calcaneal spur. Size, predisposition of foot posture, obesity, height, and BMI should be analyzed when evaluating a calcaneal spur clinically to check whether it may be associated with pain. Spur pain can be associated with size; a plantar calcaneal spur with a slope less than 30 degrees and size less than 10 mm is not typically a cause for medical concern based on prior research [2]. A high rate of calcaneal spurs have been noted to occur without causing any pain [3], leading to the conclusion that spurs might not be the sole cause of pain. Additionally, research studies have shown that in athletes, height, weight, and body mass index (BMI) do not correlate with chronic plantar heel pain [4]. As with many medical conditions, obesity can be a concern for chronic plantar heel pain given certain predispositions. Obesity and pronated foot posture correlate with chronic plantar heel pain [5]. In other words, certain factors of heel pain can influence the risk of developing heel pain while others may not be as clinically important.

Calcaneal spurs may be painful due to factors including calcaneal nerve compression, inflammation, occupational environment, spur fractures, and fat pad atrophy. They can be painful when they involve inferior calcaneal nerve compression, also known as Baxter’s neuritis or neuropathy. The inferior calcaneal nerve is located on the bottom surface of the foot and can be affected by obesity, flat feet, plantar fasciitis, muscular enlargement, foot hyperpronation, or a calcaneal spur. This condition is normally diagnosed with magnetic resonance imaging (MRI) [6]. If the calcaneal spur is causing nerve compression, then the treatment plan may vary to address the nerve compression. Occupations that may cause increased intensity of calcaneal spur pain include professions that require walking and standing for long hours [7].

Calcaneal spurs can be associated with pain following trauma to the foot or a fracture [8]. This can be diagnosed with an X-ray. Fat pad atrophy is also a cause of heel pain with a calcaneal spur. Studies have shown that patients with a thinner fat pad are at an increased risk of developing heel pain [9].

Treatment initially consists of physical therapy with exercises that include stretching, taping, and strapping stressed muscles and tendons. Shoe inserts and night splints are recommended. Initially, analgesia with acetaminophen and non-steroidal anti-inflammatory drugs (NSAIDs) is recommended. Corticosteroids can be used if pain persists. Most patients will improve with non-surgical treatment, but if symptoms persist for over 9-12 months, then surgery may be indicated [2]. Surgical intervention includes the release of the plantar fascia and the removal of the spur.

## Conclusions

This report highlights the case of a patient with chronic heel pain due to plantar fasciitis and a calcaneal spur. Plantar fasciitis and calcaneal spurs can cause significant chronic heel pain but can be improved with relatively simple measures. A thorough understanding of the anatomy, common symptoms, and treatment for this disorder will aid in reducing morbidity.
